# Implications of the COVID-19 pandemic for patients with schizophrenia spectrum disorders: narrative review

**DOI:** 10.1192/bjo.2020.157

**Published:** 2021-01-12

**Authors:** Naista Zhand, Ridha Joober

**Affiliations:** Schizophrenia and Recovery program, The Royal Ottawa Mental Health Centre, Canada; Douglas Mental Health University Institute, McGill University, Canada

**Keywords:** COVID-19, psychosis, schizophrenia, serious mental illness

## Abstract

**Background:**

COVID-19 was declared a pandemic in March 2020, by the World Health Organization. The pandemic has had unprecedented worldwide implications, in particular on marginalized populations.

**Aims:**

The aim of this study is to review the impact of the pandemic on patients with schizophrenia spectrum disorders.

**Method:**

A number of databases were searched for this review, including PubMed, EMBASE, PsycINFO and Google Scholar. Search terms included psychosis and COVID-19, schizophrenia and COVID-19, and severe mental illness and COVID-19. We included all English language papers and preprints. The final search was done on 15 July 2020.

**Results:**

Forty-seven relevant studies were identified and included in this review. Studies were summarised into five main subcategories: potential impact of the COVID-19 pandemic on physical health outcomes of patients with schizophrenia spectrum disorders, impact on mental health outcomes, review of case reports and case series to date, treatment recommendation guidelines and risk of increased prevalence of psychosis.

**Conclusions:**

Patients with schizophrenia spectrum disorders may be vulnerable to the effects of the COVID-19 pandemic. This patient population has a number of risk factors, including psychosocial adversities and illness related factors. Continuous monitoring and long-term studies of the impact of the pandemic on this patient population are required.

The COVID-19 pandemic has had unprecedented worldwide implications not only for health, but also for many aspects of life, including the economy, employment, education and family tradition. The fear and stress associated with the pandemic itself, as well as measures to confine the spread (i.e. physical distancing, societal restrictions and closures of many activities), have resulted in increased societal stress to the point that some experts postulate that mental illness will be the next inevitable pandemic.^1^ In general, disasters have a disproportionate impact on vulnerable and marginalised populations. Among these are patients with schizophrenia spectrum disorders (SSD), who are purportedly unduly affected by the current pandemic.^2^ In this narrative review, we aim to summarise all of the relevant publications, to date, on the implications of the pandemic for patients with SSD.

## Method

This is a narrative review, with the aim of providing a summary of the studies on the impact of the COVID-19 pandemic on patients with schizophrenia spectrum and related disorders. Considering the rapidly evolving and multifaceted nature of this review, we used broad inclusion criteria. The database searched for this review were PubMed, EMBASE, PsycINFO and Google Scholar. Search terms included ‘psychosis and COVID-19’, ‘schizophrenia and COVID-19’, and ‘severe mental illness and COVID-19’. We included all English language papers and preprints that reported on any COVID-19-related topics in schizophrenia spectrum and related disorders. SSD were defined using the DSM-5^3^ classifications, which include delusional disorder, brief psychotic disorder, schizophreniform disorder, schizophrenia, schizoaffective disorder, substance/medication-induced psychotic disorder, psychotic disorder owing to another medical condition and other specified and unspecified SSD.

We also searched studies of severe persistent mental illness (SMI), because SSD is one of the main subcategories of SMI. SMI studies were included if there was a clear relevance to the SSD population (i.e. risk factors, health services, etc.). We included case reports, case series, case–control studies, commentaries, viewpoints, guidelines and letters to the Editor. Considering the increasing number of publications on this topic, we updated our initial search during the process of writing this paper. The results are reported in a separate subheading, summarising studies relevant to each section.

## Results

Forty-seven relevant studies were identified and included in this narrative review. There were no randomised controlled trials on this specific topic. The majority of the included studies were viewpoint/expert opinion, case report/case series, case–control studies and treatment guidelines. We summarised the findings of these studies under five main headings: impact of the COVID-19 pandemic on the physical health of patients with SSD, impact of the COVID-19 pandemic on mental health outcomes in this patient population, a summary of case reports and case series, practice guidelines relevant to management of patients with SSD during the pandemic, and risk of increased prevalence of psychosis. Supplementary Table 1 available at https://doi.org/10.1192/bjo.2020.157 provides a summary of characteristics of the included studies in this review.^2,4-50^

### Potential impact of the COVID-19 pandemic on physical health outcomes of patients with SSD

#### Risk factors for COVID-19 infection and poorer outcome

Patients with SSD are postulated to be at a higher risk of acquiring COVID-19 and having a poorer health outcome.^2,4^ The increased risk of poorer health outcomes are attributed to factors such as higher rates of disadvantageous lifestyle, residential instability and smaller social networks.^5,6^

Patients with schizophrenia and related disorders are more likely to be over represented in congregated living situations, which puts them at additional risk for COVID-19 infection. Moreover, deficits in cognition and judgement among people with SSD may interfere with implementation of recommended measures such as hand washing, physical distancing, etc.^2,4,47^ Patients with SSD are reported to be at potentially higher risk of developing respiratory infections, in particular in the presence of other underlying medical conditions and lifestyle risk factors.^7^

Furthermore, patients with SSD have high rates of smoking and medical comorbidities such as cardiovascular disease, diabetes and respiratory illnesses; all of which are known to be associated with poorer prognosis in people who acquire COVID-19 infection.^2,8,47^ This population is also reported to have higher rates of impaired lung function, and higher rates of intensive care unit (ICU) admissions and morbidities when admitted to hospital for pulmonary conditions.^7^ Additionally, patients with SSD are generally more likely to experience disparities in accessing primary care, and are more likely to have undiagnosed or untreated underlying medical conditions.^2,9^ Other potential risk factors include those related to treatment of SSD itself, namely increased risk of hypersalivation and aspiration as a side-effect of treatment with some antipsychotics as well as, risk of toxicity during acute medical illness.^2^

Higher risk of coagulopathy is another consideration for patients with SSD who acquire COVID-19 infection.^10^ COVID-19 has been associated with hypercoagulation, and current guidelines recommend routine use of thromboprophylaxis in patients with COVID-19 infection. At the same time, patients with SSD have been reported to have a two- to three-fold increased risk of deep vein thrombosis or pulmonary embolism,^51^ likely multifactorial in nature, including biological dysregulation of the illness itself, medications and lifestyle. As such, it is hypothesised that patients with SSD who contract COVID-19 may be at higher risk of thrombotic complications, considering that both illnesses increase such risk.^10^

In addition to health risks related to COVID-19 infection in patients with SSD, there is concern about worsening of underlying medical conditions as a result of lack of access during the pandemic.^11^ In general, patients with SMI, including patients with SSD, have high rates of chronic medical comorbidities and higher rates of undertreated conditions compared with the general population. Restrictions on access to ‘non-urgent’ care during the COVID-19 pandemic may result in further disruption to physical care and/or worsening of the untreated underlying medical condition in this patient population.^11^

#### Strategies to increase health support for patients with SSD

A number of strategies were suggested to help increase health support for patients with SMI, including those with SSD. Some of the proposed strategies included providing up-to-date information tailored toward this particular patient population.^5,6^ Moreover, additional support to maintain healthy lifestyle habits and continuation of required care for underlying medical conditions were recommended.^6^

Other authors suggested an individualised approach to patient education, to increase adherence with infection control measures.^47^

It is also recommended that mental health services provide clear direction and remote contact channels to ensure continuity of care and reduce the risk of exposure. Continuous support for adherence with medication and the consideration of medication delivery are also suggested.^7^

### Mental health outcomes: potential impact on course and treatment

#### Impact of the pandemic and restrictions

There is an increasing concern about the impact of the current pandemic on the mental health of the general population, and in particular, on patients with pre-existing mental illnesses. It is hypothesised that a number of factors may lead to worsening of psychosis in patients with SSD; these factors include fear and stress caused by the pandemic, infection with the virus, distress and isolation among those who acquire the virus, and treatment of the viral illness with steroids and other agents.^2^

Furthermore, the societal restrictions of the pandemic may have a negative impact on patients with SSD. In general, patients with SSD have smaller social networks, and therefore, further restricting their already tenuous support may be associated with negative consequences. Moreover, social isolation has been associated with poorer quality of life and paranoia,^12^ as well as being a risk factor for suicide among patients with SSD. A potential increase in substance use during social isolation has also been presumed,^12^ which could lead to further deterioration in this patient population. As such, maintaining connectedness through virtual means, as well as continuation of essential community services, is paramount for this patient population.^2^

Another consideration is the economic burden of the pandemic on this patient population. Patients with SSD are more likely to have jobs without sick-leave or benefits, and are more vulnerable to loss of employment.^4^

In light of reports from the Swine flu outbreak, Hamada and Fan^12^ propose potential worsening of symptoms of obsessive–compulsive disorder, a common comorbidity in patients with SSD. Fears regarding acquiring infection may result in increased obsessive thoughts, which could add to functional limitations associated with SSD.

The ‘infodemic’, a surfeit of information about COVID-19 infection, has also been argued to potentially cause exacerbation of psychotic symptoms.^12^ Reduction of media exposure has been suggested by some authors, with the rationale that overexposure to broadcasts of stressful situations has been associated with negative mental health outcomes.^7^

Iasevoli et al^13^ interviewed 205 patients with SMI 1 month after quarantine in Italy, and compared this group to 205 controls and 51 relatives of patients. Their results showed patients with SMI were four times more likely to perceive higher stress related to the pandemic than controls, and had two to three times higher risk of significant anxiety and depressive symptoms. They reported similar outcome when they separated patients with schizophrenia from those with mood disorders.

#### Changes in care delivery

Uninterrupted access to psychiatric care is imperative for patients with SMI, including those with SSD, to prevent decompensation.^14^ During the COVID-19 pandemic, many out-patient services have been replaced by virtual means, such as telephone check-ins and telepsychiatry visits. On the other hand, many services have been cancelled, such as day programmes and peer support groups.^4,12^ Although the nature of the pandemic and health risks of COVID-19 infection require such measures, the impact of changes in mental healthcare delivery in this patient population has yet to be examined.^2^

Torous and Keshavan^15^ discussed the need for mental health support for patients with SSD (and patients with SMI in general) during the pandemic, and suggested the use of smartphones as an effective way to implement telepsychiatry. They discussed the potential for implementation of apps in the clinical care of patients with SSD, including creating personalised care plans, monitoring symptoms and relapse prediction.

In addition to changes to out-patient services, access to some of the evidence-based community services, such as assertive community treatment and case management, might become limited or replaced by virtual methods.^2^

Like many other services, hospital-based and in-patient psychiatric care have undergone changes during the COVID-19 pandemic. To date, not much is known about the potential impact of these necessary changes. For instance, following major outbreaks in the in-patient psychiatric units of Wuhan and South Korean hospitals, psychiatric wards have embraced preventative measures, such as prohibition of visitors and external food/clothing,^2^ or total lockdown of the in-patient units.

Other care delivery changes include laboratory requirements, monitoring of patients taking clozapine and access to long-acting injectable (LAI) antipsychotic medications for patients with SSD. Although clozapine monitoring guidelines have proposed exceptions to the regular blood monitoring requirements (see the guideline section), patients who are in the first year of treatment with clozapine still require weekly or biweekly blood work, which may put them at increased risk of exposure. During the pandemic, regulatory agencies such as the USA Food and Drug Administration have implemented clozapine-monitoring programmes, allowing for a ‘grace period’ of up to 56 days for clozapine to be dispensed without blood work. However, when contacted, one pharmacy in a small community indicated they would only dispense up to 2 weeks of clozapine if a patient was late for blood work.^16^ The authors highlighted the need for a collaborative approach to ensure continued care for patients taking clozapine.

At the same time, restricted access to hospitals under the advice of health authorities may create a challenge for patients who are on LAI antipsychotics. Ifteni et al^17^ reported on data from a psychiatric hospital in Romania. Their results revealed a 49% reduction in the use of risperidone LAI antipsychotics, and a 70–90% reduction of aripiprazole, paliperidone and olanzapine LAI medication use in March 2020, compared with the 3-month period before the outbreak. Of concern is the increased risk of non-adherence and relapse subsequent to switching an LAI medication to oral treatment.

Nichols et al^18^ reported on changes implemented in their clozapine clinic, which serves 184 patients, during the pandemic. They shifted in-person nursing assessments to telepsychiatry visits, started to deliver clozapine for curb-side pick-up or through the mail, and implemented blood work exemptions (up to 3 months) as per USA Food and Drug Administration recommendations. These strategies significantly reduced the in-person visits, and therefore the risk of transmission of COVID-19.

Grover et al^19^ reported on the use of telecommunication to monitor patients receiving clozapine in an under-resourced area in India. These telephone appointments included information on symptoms of COVID-19, when to present to an emergency department and education regarding continuation of clozapine and regular blood work monitoring. Through these telephone calls, they were able to reach 205 out of 227 patients on clozapine. Of those contacted, 81.5% were in touch with their treating physician. Overall, 96.6% of patients stayed on the same dose of clozapine; however, about one fourth had difficulty obtaining clozapine because of a lack of availability at their local pharmacy. Only 24.4% were able to have blood work done in the previous month. The authors highlighted the importance of telephone monitoring of patients on clozapine in under-resourced areas. At the same time, this study also draws attention to the disproportionate impact of the pandemic on patients with SMI, including those with SSD, and in particular, those in lower socioeconomic areas.

Kopelovich et al^20^ described changes in healthcare delivery across the USA, and made suggestions to better align these changes with current standards of practice. They discussed the use of multimodal service delivery, including a combination of telehealth, in-person clinics, community outreach, other digital interventions and warm lines. Warm lines were described as support lines for patients with no family or other sources of support. Such a multimodal service plan will provide flexibility based on clients’ needs and barriers to care. The authors also recommended development of COVID-19-compatible advance directives. Further recommendations included assessment through telehealth visits and encouraging clinicians to adapt to the patient population. For instance, patients with psychosis may not be able to tolerate a lengthy visit over telehealth, and in such cases, shorter but more frequent visits are suggested. For patients with SMI, and in particular patients with SSD, it is better for the first assessment to be done in person, considering the importance of building rapport, which could be challenging over telehealth, especially for patients with paranoia or disorganisation. They also recommended a careful risk–benefit assessment when deciding on the mode of care delivery for clients at elevated safety risks (i.e. suicide/violence). In certain cases, prioritisation for in-person visits is essential for management of psychiatric morbidity. Other recommendations include consideration of telepsychotherapy to address anxiety and insomnia, management of substance use, and addressing primary care and medication needs.

### Review of case reports and case series to date

#### Case reports

To date, a number of case reports have been published on both new onset of psychotic symptoms and worsening of pre-existing psychosis in the context of COVID-19 infection. Of the ten case reports that we found, two studies each reported on a case with no previous psychiatric history and a brief psychotic episode triggered by the stress of the pandemic.^21,22^ There were three case reports with new-onset non-reactive psychosis: one in a patient self-medicated with chloroquine;^23^ one in a patient who had been recently admitted to hospital for COVID-19 infection and received treatment including hydroxychloroquine and prednisone;^30^ and one in a patient who was admitted for COVID-19 infection and developed psychosis requiring a second admission to the hospital.^31^ Three studies reported a relapse of psychosis triggered by the stress of the pandemic^35,36^ or quarantine.^37^ In one of these cases, the patient had incorporated COVID-19 into their delusional system, and developed symptomatic COVID-19 infection during their hospital stay;^36^ and in another case, the patient was found to have a positive COVID-19 test result.^37^ The two remaining case reports were also about patients with pre-existing psychotic illness and COVID-19 infection: one presented with relapse of psychosis and delirium^39^ and one presented with clozapine toxicity.^40^

In a Letter to the Editor, de Burgos-Berdud et al^24^ reported on four studies that described reactive psychosis or psychotic symptoms triggered by the COVID-19 pandemic in a total of eight healthcare workers. They highlighted the need for providing support for healthcare workers during the pandemic.

#### Case series of reactive psychosis or psychotic relapses

Martin et al^32^ reported on a case series from Spain (with an unknown number of cases) that had no previous psychiatric history and presented with COVID-19 infection and acute psychosis. They described subacute onset (less than a week) and fast recovery (within 2 weeks) in response to low-dose antipsychotic treatment.

In Italy, Finatti et al^25^ described three cases without any previous psychiatric history, who presented with brief psychotic disorder in the context of social isolation during the COVID-19 pandemic.

In another case series study from Spain, Valdés-Florido et al^26^ reported on four cases of brief psychotic disorder during the first 2 weeks of national quarantine. Two of the cases presented with serious suicidal behaviour. The authors discussed a potential rise in cases of reactive psychosis as a result of the COVID-19 pandemic.

Shanbour et al^27^ presented three cases with first-episode psychosis and paranoid delusions explicitly about COVID-19. One individual had delusions about the cell phone tower beside his apartment spreading COVID-19, another individual believed she was pregnant and that her negative pregnancy tests were because of COVID-19 infection, and another individual had delusions about being God's son and that he had predicted the COVID-19 pandemic 3 years prior. The authors highlighted the impact of the pandemic, the quarantine and infodemics on the precipitation of psychosis in vulnerable individuals.

Chandra et al^28^ reported on two cases from India presenting with psychosis related to COVID-19. Both cases were precipitated by fear of acquiring COVID-19, on a background of personal vulnerabilities interfacing with sociocultural issues. The authors discussed COVID-19 as a social illness resulting in high stress, in particular in vulnerable individuals.

Ferrando et al^33^ reported on three cases, all of individuals aged 30–39 years, who presented with new-onset psychosis and were found to have a COVID-19 infection that was otherwise asymptomatic. They had elevated inflammatory markers, and particularly C-reactive protein. None of these cases were overtly preoccupied or worried about COVID-19 infection. All cases were treated with low-dose antipsychotics. The authors discussed potential immune-mediated psychopathology in these cases, and drew attention to ‘COVID-19 psychosis’ as a potential complication of COVID-19, requiring further exploration.

In Spain, Parra et al^34^ reported on a retrospective study of ten patients with COVID-19 infection who developed psychotic symptoms. They excluded delirium cases. The mean age was 54.1 years. Only one case had no physical manifestation of COVID-19 infection, and their presentation was purely psychiatric. In 80% of cases, the onset of psychotic symptoms occurred 2 weeks after the initial symptoms of COVID-19, and these symptoms resolved in less than 2 weeks, with low-dose antipsychotics. The most frequent symptoms included structured delusions and confusion, although the latter could be explained in the context of medical illness (80% had bilateral pneumonia) and ICU treatment (50% of this sample). The authors discussed possible mechanisms of COVID-induced psychosis, including direct central nervous system involvement, indirect effect through neuroinflammation and metabolic disturbance, and iatrogenic disease via medications for treatment of COVID-19 and its complications.

#### Case series of patients with SSD with suspected COVID-19 infection

Liu et al^38^ reported on 21 in-patients with schizophrenia who were transferred to an isolation unit of a psychiatric hospital for suspected COVID-19 infection. The comparison group included 30 in-patients with schizophrenia without any COVID-related symptoms. Only one patient had positive COVID-19 test results, but another 11 patients met the diagnostic criteria for clinically confirmed cases. The Positive and Negative Syndrome Scale score was similar in both groups. However, compared with controls, patients with suspected COVID-19 infection scored significantly higher on depression, anxiety, perceived stress and sleep quality scales. About half of the patients with suspected COVID-19 (52.4%) required an increase in dose or addition of medications for management of their psychiatric symptoms.

#### Psychotic experiences in students

Hajdúk et al^29^ assessed psychotic experiences among students from Comenius University in Bratislava, using surveys 1 year before and during the initial phase of the COVID-19 pandemic. There were no significant changes in the scores across the study time points. However, they found a significant relationship between positive psychotic experiences and negative affectivity, in particular depressive symptoms. The authors discussed that the lack of change in psychotic experiences in this study could be explained by milder severity of the pandemic in the country or by some protective factors such as increased contact with family and higher social cohesion. Furthermore, this study was conducted at earlier phases of the pandemic and the impact on psychosis may take longer to manifest.

#### Case–control studies

In a nationwide study in Korea, Ji et al^41^ assessed comorbidities in patients who tested positive for COVID-19 compared with those with negative results. The study included 219 961 individuals who were tested for COVID-19 infection up until 15 May 2020, using reimbursement data and the KCD-7 diagnostic codes. Use of respiratory support was considered as a proxy measure for severity of COVID-19 infection. In patients with COVID-19 infection, comorbidities with significant association included diabetes (odds ratio range 1.2), osteoporosis (odds ratio range 1.1), rheumatoid arthritis (odds ratio range 1.2), substance use (odds ratio range 1.3) and schizophrenia (odds ratio range 1.6). Comorbidities associated with severe COVID-19 were all medical (i.e. diabetes, end-stage renal disease, etc.) and non-psychiatric. The authors concluded that the higher rate of comorbidity with schizophrenia is likely a result of outbreaks in psychiatric units or group homes rather than the vulnerability caused by the illness itself.

### Treatment recommendation guidelines

A consensus statement on the use of clozapine during the COVID-19 pandemic was published by an expert advisory subgroup of the Treatment Response and Resistance in Psychosis Working Group.^42^ The purpose of the statement is to provide guidance for clinicians and regulatory agencies to ensure continued access to clozapine during the pandemic. The statement recommends reduction of blood work monitoring to every 3 months for patients who have been on clozapine for over a year and have never had an absolute neutrophil count <2000/mL. They also recommend urgent physician assessment for patients on clozapine who develop any signs of infection; the rationale behind this suggestion is an increased risk of pneumonia owing to sialorrhea and risk of aspiration in patients taking clozapine. Finally, the statement recommends to monitor patients taking clozapine who develop fever and flu-like symptoms, for symptoms of clozapine toxicity. Considering acute systematic infections can increase clozapine levels, the statement suggests reduction of the clozapine dose until 3 days after resolution of symptoms, if symptoms of clozapine toxicity emerge.

The Croatian Society for Schizophrenia and Schizophrenia Spectrum Disorders^43^ also released guidelines for treatment of patients with SSD during the COVID-19 pandemic. They provided a table of possible drug interactions between selected antivirals, antibiotics and common psychiatric medications. They recommended continuation of patients’ regular medication regimen, adherence to COVID-related social restrictions and telephone consultation with the psychiatrist, if needed.

In the UK, Gee et al^44^ published a more detailed guideline on management of clozapine treatment during the COVID-19 pandemic. They recommended increasing the interval of blood work to every 12 weeks in patients treated with clozapine for over a year. In those who present with symptoms of COVID-19, they suggested rapid antigen testing as well as complete blood count testing, to distinguish COVID-19-related symptoms from side-effects of clozapine. They recommended continuation of clozapine in patients with COVID-19 infection, and only reducing the dose if necessary, preferably as guided by plasma levels of clozapine. Weighing risks versus benefits, the authors recommended continuation of clozapine in ICU settings and during periods of sedation, with careful monitoring. Furthermore, they suggested focusing on neutrophil count rather than on total white blood cell count, as COVID-19 is linked to lymphopenia. Another recommendation was to initiate vitamin D supplementation in all patients taking clozapine, considering the high rates of low vitamin D in patients with schizophrenia and some possible protective effects in decreasing the likelihood of COVID-19 respiratory infection.

### Risk of future development of psychosis

In addition to the short-term implications, it has been postulated that the COVID-19 pandemic may result in an increased prevalence of psychosis in the coming decades.^45^ The authors discussed a number of potential explanations as to how the current viral pandemic may influence the risk of developing schizophrenia. The association between *in utero* exposure to viral illnesses and development of schizophrenia has been considered for years. Although this theory remains controversial,^52^ an observational study by O'Callaghan et al^53^ showed an increased rate of schizophrenia in children born 5 months after the peak of the flu pandemic in 1957. Severance et al^54^ examined the antibodies of four strains of coronavirus in patients with a recent psychotic episode. Compared with controls, patients with recent psychosis had significantly elevated coronavirus antibodies, suggesting an association between coronavirus infections and psychosis. Other potential mechanisms discussed include direct invasion of the virus into nerve cells, activation of autoimmune processes by the virus, and genetic modification of the immune system by viruses and subsequent negative impact on the central nervous system.^45^

In China, Hu et al^46^ reported an observation of increased incident diagnosis of schizophrenia in an out-patient setting in January 2020, which correlated with the timeline of COVID-19 in China. They used data on first-onset schizophrenia for the month of January from three consecutive years (2017–2019) as a comparison. Their results showed a 25% increased rate of diagnosis of schizophrenia in first-time patients, and the risk was higher among the 39–50 year age group. This observation was reported from a province with minimal COVID-19 infection in January (23 COVID-19 cases in a population of 8 million). This study is included in our review for comprehensiveness, considering our ever-changing understanding of COVID-19; however, the reported observation of new-onset schizophrenia and correlation with COVID-19 in this study is questionable. This study was published on an open platform (not a peer-reviewed journal), and one of the major concerns of this study is the fact that by definition, a diagnosis of schizophrenia requires 6 months of symptoms, and therefore, by default, the onset of these new diagnoses were at least 6 months before the study (and COVID-19) timeline.^46^

Brown et al^47^ reviewed studies of previous viral epidemics and pandemics. Their results showed a rate of 0.9–4% for psychosis in people infected by a virus, which is much higher than a median incident rate of psychosis of 15.2 per 100 000. The limited evidence suggested that patients who developed psychosis in the context of a viral exposure responded to treatment with low-dose antipsychotics. On the other hand, a systematic review and meta-analysis by Rogers et al,^48^ of psychiatric presentations specifically associated with previous coronavirus outbreaks, showed only a small percentage of psychosis or mania (0.7%). Almost all of these patients, who were infected with severe acute respiratory syndrome or Middle East respiratory syndrome, had received corticosteroids.

One study by Varatharaj et al^49^ presented data from a UK-wide surveillance study of neurological and psychiatric complications of COVID-19 infection in 153 patients. They launched an online platform and encouraged physicians to report cases meeting each classification definition (i.e. cerebrovascular events, altered mental status, etc.). During the 24-day study period, the system received notifications regarding 153 cases, with complete data available for 125. The median age of patients was 71 years and there were no cases under 20 years. A total of 62% presented with cerebrovascular events, and 31% presented with altered mental status. Overall, 23 out of 125 patients were classified as having psychiatric or neuropsychiatric complications; of those, 43% had a new-onset psychosis diagnosis (7.9% of the total 125 cases). As discussed by the authors, these numbers are only a snapshot of patients admitted to hospital with moderate-to-severe COVID-19 infection with psychiatric complications, and future prospective studies are required to adequately determine the extent of the psychiatric sequelae of this pandemic. Furthermore, not much is known about potential neuropsychiatric complications of COVID-19 in younger populations, which requires further study.

On the other hand, Vukojević et al^50^ discussed their view on potential protective effect of the pandemic against psychosis. They reported no increase in psychiatric or psychosis-related admissions over the course of the pandemic so far, even after a significant earthquake occurred during the same time in Croatia, resulting in substantial damage. They noticed a similar pattern of no change in psychiatric admission rates four months after the Croatian War of Independence in the 1990s. They proposed the contagion psychology theory as a possible explanation to these observations. This theory proposes that people unconsciously mirror emotion and behaviour of the crowd, and eventually experience similar feelings and behaviours. This effect is more prominent, as the uncertainty and stress of the event increases. The authors hypothesised that the collective effect of the pandemic results in keeping society stable or ‘sane’. They combined the contagion theory with an evolutionary perspective, which considers psychosis as a defence mechanism; they explained that staying at home with close family, as opposed to being exposed to a changing environment (owing to COVID-19), may reduce the psychological or subconscious need of manifestation of psychosis.

## Discussion

The present paper reviews the current state of knowledge on the impact of the COVID-19 pandemic on patients with SSD. As discussed by several studies, patients with SSD have several risk factors, including health-related and socioeconomic risks, potentially putting them at higher likelihood of negative consequences. There are a number of case reports and case series that described relapse of psychosis in this population and/or new-onset psychosis in patients without any previous history. One of the key messages discussed by many articles in this review is the importance of continuity of psychiatric care and treatment during the pandemic. Many services have switched over to virtual care delivery successfully; however, in caring for patients with SSD, an individualized approach and some flexibility may be required when use of virtual methods are not feasible or sufficient.

Although not discussed by the reviewed papers, the authors would like to draw attention to an ethical issue that arises from long hospital stays without visits and, therefore, infringements on the rights of these patients. Although restricting out-of-hospital passes and visitors are necessary steps to control the spread of the virus, this becomes more problematic during the lengthy hospital stays seen in patients with SSD.

At the time of writing this paper, we are 4 months into the pandemic, and so far, our knowledge of the impact of the pandemic on this patient population remains mostly speculative and based on known risk factors or evidence from previous outbreaks. Future epidemiological studies will help to shed light on the longer-term impact of the COVID-19 pandemic.

There are a number of limitations of this review paper, such as inclusion of preprints, studies with small sample sizes and the relatively weak methodology of the majority of the included papers. Such limitations are a result of the rapidly evolving nature of this pandemic, and the evolution of studies as we learn more about the COVID-19 pandemic and its impact.

## Supplementary material

For supplementary material accompanying this paper visit https://doi.org/10.1192/bjo.2020.157. click here to view supplementary material
