# Supplementary material for: Implications of the COVID-19 pandemic for patients with schizophrenia spectrum disorders: narrative review
Source: BJPsych Open. 2021 Jan 12;7(1):e35. doi: 10.1192/bjo.2020.157 (PMC7804069; doi:10.1192/bjo.2020.157)
Supplement: Supplementary file 1 [file S205647242000157Xsup001.docx]

Supplementary Table 1 Summary of the included studies

|  | Author(s) | Country | Journal | Study/article type | Summary |
| --- | --- | --- | --- | --- | --- |
| **1. Impact of the covid-19 pandemic on physical health outcomes of SSD** | | | | | |
| 1 | Kozloff et al^2^ | Canada | Schizophr Bull | Expert opinion/review | The authors discuss the potential negative impact of the COVID pandemic on health, mental health and management of SSD |
| 2 | Shinn et al^4^ | United States | J Clin Psychiatry | Expert opinion/review | The authors discuss the higher risk of acquiring COVID, poorer outcomes as well as the negative impact of the public health response and changes in care delivery in patients with SMI (and SSD) |
| 3 | Maguire et al^5^ | Australia | *Aust N Z J Psychiatry* | Letter | The authors discuss the vulnerability of patients with schizophrenia to COVID-19 due to factors such as medical comorbidities, life style, poor engagement with health care system, and treatment with antipsychotics. |
| 4 | Druss^6^ | United States | JAMA Psychiatry | Expert pinion/ View point | The author provide suggestions to address the pandemic in SMI populations in terms of supporting patients, empowering clinicians and strengthening care systems |
| 5 | Fonseca et al^7^ | Brazil | Braz J Psychiatry | Expert pinion/ View point | The authors discuss the health impact of COVID-19 on patients with schizophrenia and provide recommendations |
| 6 | Bartels et al^8^ | United States | Psychiatr Serv | State mental health policy | The authors discuss disparities in health and the increased risk of morbidity and mortality due to COVID-19 in patients with SMI including schizophrenia. They provide a highlight of the COVID-response in the state of Massachusetts in this patient population |
| 7 | Kahl and Correll^9^ | Germany | JAMA Psychiatry | Expert pinion/ View point | The authors discuss strategies to improve health support for SMI patients including patients with schizophrenia. |
| 8 | Mongan et al^10^ | Ireland | Brain Behav Immun | Expert pinion/ View point | The authors discuss the potentially increased risk of hypercoagulation in patients with SSD |
| 9 | Melamed et al^11^ | Canada | Gen Hosp Psychiatry | Editorial | The authors discuss disparities in chronic physical disease, accessing health care among SMI patients and mitigation strategies |
| **2.Impact of Covid-19 pandemic on Mental Health outcomes of SSD**^a^ | | | | | |
| 10 | Hamada and Fan^12^ | United States | Schizophr res | Expert pinion/ View point | The authors discuss challenges and the risk of decompensation in SMI patients including patients with SSD, and mitigation strategies |
| 11 | Iasevoli et al^13^ | Italy | Psychol Med | Case control | This study compares the psychological impact of the COVID pandemic and quarantine among: 205 patients with SMI (& SSD) >205 controls >51 caregivers |
| 12 | Hategan and Abdurrahman^14^ | Canada | Psychiatry Clin Neurosci | Expert pinion/ View point | The authors discuss the needs of high-risk psychiatric populations during the COVID-19 pandemic, including the need for uninterrupted access to services for SMI patients. |
| 13 | Torous and Keshavan^15^ | United States | Schizophr Res | Editorial | The authors discuss the use of mobile health during the pandemic for patients with SSD (& SMI patients in general) |
| 14 | Leung and Wittenberger^16^ | United States | Schizophr Res | Letter to the Editor | The authors discuss treatment with clozapine during the pandemic and ensuring continuity of care |
| 15 | Ifteni et al^17^ | Romania | Schizophr Res | Letter to the Editor | The authors report a significant drop in prescription of long acting injectable antipsychotics (LAIs) in their hospital during the COVID-19 pandemic and discuss the need for continuation of treatment with LAIs |
| 16 | Nichols et al^18^ | United States | Schizophr Res | Expert pinion/ View point | The authors discuss the need and strategies to ensure care for patients with SSD on clozapine during the pandemic |
| 17 | Grover et al^19^ | India | Schizophr Res | Letter to the Editor | The authors report on phone follow ups of 227 clozapine patients in a resource-poor setting in India |
| 18 | Kopelovich et al^20^ | United States | Community Ment Health J | Expert pinion/ View point | The authors present the observed changes in health care delivery to SMI patients (including patients with SSD) during the pandemic and provide suggestions to align these changes with best practices |
| **3.Case reports and case series** | | | | | |
| ***3.1. No previous diagnosis of psychosis and COVID negative*** | | | | | |
| 19 | Huarcaya-Victoria et al^21^ | Peru | Psychiatry Res | Case report | A case report of a brief psychotic disorder in a patient with anxiety related to COVID-19 |
| 20 | Zulkifli et al^22^ | Malaysia | Malaysian Journal of Psychiatry | Case report | A case report of a brief psychotic disorder in the context of the COVID-19 outbreak in a patient without previous history of psychosis |
| 21 | Akkaoui et al^23^ | France | Biol Psychiatry | Case report | A case report of first episode psychosis in a patient self-medicated with chloroquine |
| 22 | de Burgos-Berdud et al^24^ | Spain | Gen Hosp Psychiatry | Case report | The authors provide a summary of four studies that reported reactive psychosis among health care workers during the COVID-19 pandemic |
| 23 | Finatti et al^25^ | Italy | Prim Care Companion CNS Disord | Case-series | A case series of three patients with brief psychotic disorder in the context of social isolation during the COVID-19 pandemic |
| 24 | Valdes-Florido et al^26^ | Spain | Revista de Psiquiatría y Salud Mental | Case-series | A case series of four patients with brief psychotic disorder in the first two weeks of national quarantine |
| 25 | Shanbour et al^27^ | United States | Prim Care Companion CNS Disord | Case-series | A case series of three cases with first episode psychosis and paranoid delusions specifically related to the COVID-19 pandemic |
| 26 | Chandra et al^28^ | India | Psychiatry Res | Case-series | A report on two cases presenting with psychosis, precipitated by high anxiety about acquiring COVID-19 |
| 27 | Hajduk et al^29^ | Slovak republic | Schizophr Res | Survey study | A survey study of 235 non clinical college student, comparing the changes in psychotic experiences before and during the initial phase of the pandemic |
| ***3.2. No previous diagnosis of psychosis and COVID positive*** | | | | | |
| 28 | Correa-Palacio et al^30^ | Spain | Psychiatry Res | Case report | A case report of affective psychosis following COVID-19 infection |
| 29 | Majadas et al^31^ | Spain | Psychiatry Clin Neurosci | Case report | A case report of a patient with psychotic disorder as a clinical presentation of infection with COVID-19 |
| 30 | Martin et al^32^ | Spain | Psychiatry Res | Case-series | A case series of new onset psychosis in patients with COVID-19 infection |
| 31 | Ferrando et al^33^ | United States | Psychosomatics | Case-series | A report of three cases with new onset psychosis and asymptomatic COVID-19 infection. |
| 32 | Parra et al^34^ | Spain | Psychiatry Res | Case-series | A retrospective review of ten patients with COVID-19 infection who developed psychosis |
| ***3.3. Known diagnosis of SSD and COVID negative*** | | | | | |
| 33 | Fischer et al^35^ | Germany | Psychiatry Res | Case report | A case report of a patient with schizophrenia presenting with relapse and paranoia related to COVID-19 |
| 34^b^ | Ovejero et al^36^ | Spain | Schizophr Res | Case report | The authors present a case report of a bipolar patient with COVID-19 delusion. They discuss how COVID-19 might become a new delusional theme in psychotic disorders |
| 35 | Sanchez-Alonso et al^37^ | Spain | Psychiatry Res | Case report | A case report of a patient with schizoaffective disorder who experienced relapse as a result of quarantine |
| 36^c^ | Liu et al^38^ | China | Gen Psychiatry | Case control | A retrospective review of 21 hospitalized patients with SSD requiring isolation for suspected COVID-19 infection in comparison to a control group of 30 patients with SSD on the inpatient unit |
| ***3.4. Known diagnosis of SSD and COVID positive*** | | | | | |
| 37 | Palomar-Ciria et al^39^ | Spain | Psychiatry Res | Case report | A case report of a patient with schizophrenia who presented with delirium in the context of COVID-19 infection |
| 38 | Cranshaw and Harikumar ^40^ | United Kingdom | Schizophr Bull | Case report | A case report of clozapine toxicity due to COVID-19 infection |
| 39 | Ji et al^41^ | Korea | J Korean Med Sci | Case control | A nation wide survey of the impact of comorbidities on infection and severity of the COVID-19. Schizophrenia was among the comorbidities with significant association with COVID-19 infection |
| **4.Treatment recommendation guidelines** | | | | | |
| 40 | Siskind et al ^42^ | International | J Psychiatry Neurosci | Guidelines | A consensus statement on the use of clozapine during the COVID-19 pandemic published by an expert advisory subgroup of the Treatment Response and Resistance in Psychosis working group |
| 41 | Karlović and Peitl^43^ | Coroatia | Arch psychiatry Res | Guidelines | Guidelines for the treatment of patients with SSD during the COVID-19 pandemic by the Croatian Society for Schizophrenia and Schizophrenia Spectrum Disorders |
| 42 | Gee et al^44^ | United Kingdom | Ther Adv Psychopharmacol | Guidelines | A more detailed guideline on the management of clozapine treatment during the COVID-19 pandemic |
| **5.Risk of future development of psychosis** | | | | | |
| 43 | Zandifar and Badrfam^45^ | Iran | Psychiatry Res | Letter to the Editor | The authors discuss the potential increase in prevalence of schizophrenia, considering the historical evidence from the influenza epidemic |
| 44 | Hu et al^46^ | China | ChinaXiv | Observational study | The authors report an increased incidence diagnosis of schizophrenia in an outpatient setting |
| 45 | Brown et al^47^ | Australia | Schizophr res | Rapid review | Authors reviewed the literature on impact of pandemic and epidemic on incidence rate of psychosis |
| 46 | Varatharaj et al^48^ | United Kingdom | Lancet psychiatry | Surveillance study | The authors present data from a UK-wide surveillance study of neurological and psychiatric complications of COVID-19 infection in 153 patients. Approximately, 7.9% had a new onset diagnosis of psychosis |
| 47 | Vukojevic et al^49^ | Croatia | Psychiatry Res | Letter to the Editor | The authors discuss potential protective effects of the pandemic against psychosis |

a. Some of the articles cited under the previous section (i.e. impact on health) also discussed the impact on mental health.

b. Patient in this case report had diagnosis of bipolar (& not SSD). This study was included as the authors discuss the potential changes in delusional content of the psychotic patients.

c. One case in this study tested positive for COVID infection, and eleven cases fit clinical diagnostic criteria for COVID infection.
